# Smell of green leaf volatiles attracts white storks to freshly cut meadows

**DOI:** 10.1038/s41598-021-92073-7

**Published:** 2021-06-18

**Authors:** Martin Wikelski, Michael Quetting, Yachang Cheng, Wolfgang Fiedler, Andrea Flack, Anna Gagliardo, Reyes Salas, Nora Zannoni, Jonathan Williams

**Affiliations:** 1 Department of Migration, Max Planck Institute of Animal Behavior, Radolfzell, Germany; 2 Centre for the Advanced Study of Collective Behaviour, University of Konstanz, Constance, Germany; 3 Department of Biology, University of Pisa, Pisa, Italy; 4 Department of Atmospheric Chemistry, Max Planck Institute for Chemistry, Mainz, Germany; 5 Energy, Environment and Water Research Center, The Cyprus Institute, Nicosia, Cyprus

**Keywords:** Ecology, Zoology, Ecology

## Abstract

Finding food is perhaps the most important task for all animals. Birds often show up unexpectedly at novel food sources such as freshly tilled fields or mown meadows. Here we test whether wild European white storks primarily use visual, social, auditory or olfactory information to find freshly cut farm pastures where insects and rodents abound. Aerial observations of an entire local stork population documented that birds could not have become aware of a mown field through auditory, visual or social information. Only birds within a 75° downwind cone over 0.4–16.6 km approached any mown field. Placing freshly cut grass from elsewhere on selected unmown fields elicited similarly immediate stork approaches. Furthermore, uncut fields that were sprayed with a green leaf volatile organic compound mix ((Z)-3-hexenal, (Z)-3-hexenol, hexenyl acetate), the smell of freshly cut grass, immediately attracted storks. The use of long-distance olfactory information for finding food may be common in birds, contrary to current perception.

## Introduction

Finding food is central to the survival of animals and foraging ecology is critical to understanding animals in their natural habitat^1^. However, it has proven extremely difficult to determine which sensory system is predominately used to find food, and which combination of senses is employed by individual animals in their decisions to approach food sources^2^. In the aquatic realm, chemical signals or water-borne odors abound^3^, but hydrodynamic breathing currents^4^, sound or visual signals can also be used by aquatic foragers to find their prey. For terrestrial animals, odors are often used as long-distance cues particularly for predators in pursuit of their prey, but auditory and visual signals are similarly important^5^. Primates often appear to be ‘led by the nose’ in their foraging activities^6^. For all foragers, social stimuli such as the circling of vultures above a kill site enable the collective finding and use of food sources^7^.

In contrast to most aquatic and terrestrial animals, for birds, olfactory signals are only known to be used by a few select taxonomic groups to find food^8–13^. New world vultures have experimentally been shown to use scent to find carcasses^14–16^. Similarly, several groups of seabirds can use dimethyl sulfide (DMS) to home in on upwelling areas where masses of marine microorganisms as well as their vertebrate prey assemble at the sea surface, facilitating the oftentimes collective foraging of marine birds^9,17^. While the perceived knowledge of birds foraging primarily visually or auditorily has guided our studies of avian foraging ecology, it has become clear over the past decade that olfactory cues may be more important for birds than previously expected^17,18^. Avian olfactory gene repertoires are well-developed and could enable an excellent sense of smell^19^. Indications for the use of odors in birds are that starlings select nest material based on odors^20^ and songbirds may use odor to recognize and avoid the presence of their predators^8^. Partner choice may in part be influenced by odors in Zebra finches^21^ and seabirds^22^. Songbirds searching for insect larvae may use the odors excreted by ‘talking trees’^23,24^ sounding chemical ‘alarm calls’ against insect herbivory^25,26^. And navigating birds such as homing pigeons^27^, petrels^28^, catbirds^29^ or gulls^30^ appear to use aerial olfactory cues to know where they are with respect to their goal.

**Figure 1:**
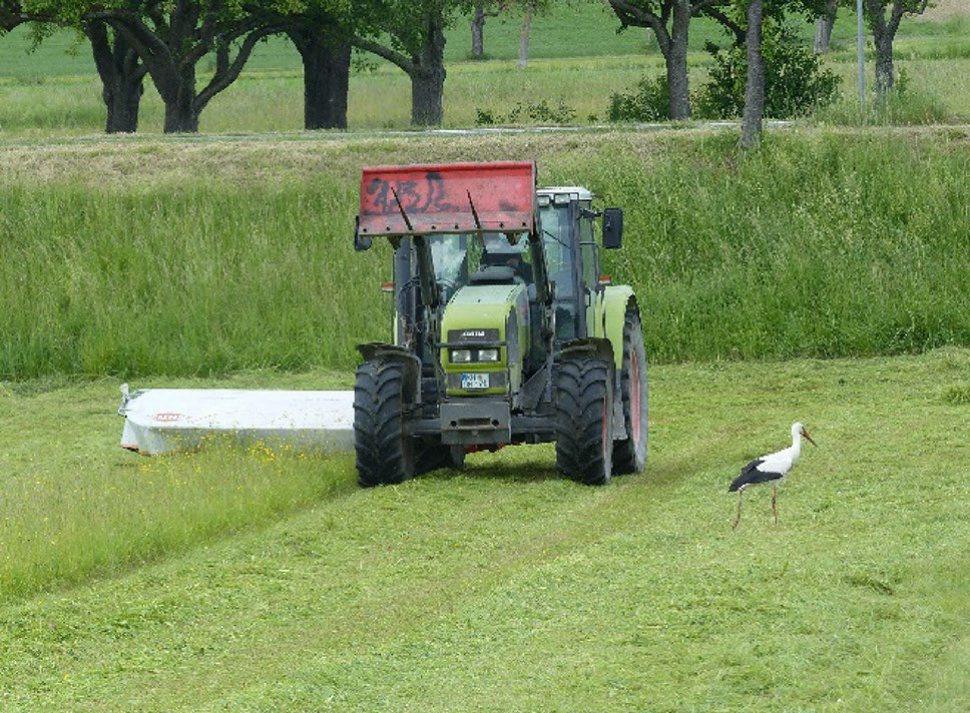
A typical observation of European white stork next to a farmer mowing tall grass. Storks use this association with humans to forage for insects or rodents and other vertebrates disturbed by farming activities. However, farmers are not always approached by storks when mowing. Photo by Christian Ziegler, MPIAB.

Here we make use of a commonly observed phenomenon to test experimentally whether birds use airborne odor cues to find food over long distances. European white storks are known to aggregate on fields where farmers cut grass (Fig. 1). It is so far unclear whether storks also approached mown fields already during pre-industrial times. Interestingly, farmers are often puzzled by the fact that storks only approach them sometimes during their mowing activities.

## Methods

### Aerial and ground observations

**Figure 2:**
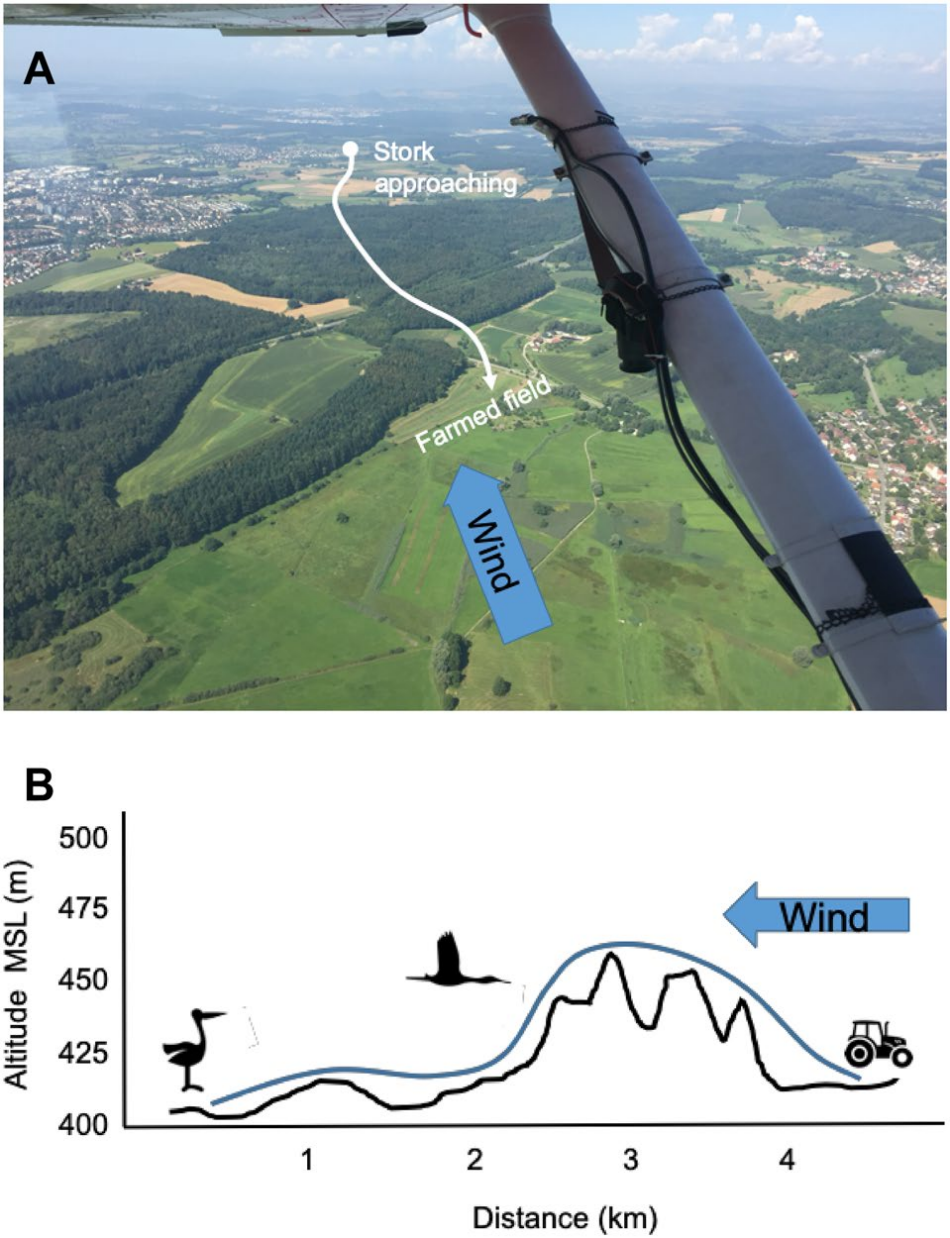
An aerial view of the observed small-scale farming landscape around Lake Constance, Southern Germany and a detailed example of a stork approach to farming activity. (**A**) About 1 h after a farmer had starting mowing activities (in the foreground), the aerial information at a windspeed of 4 km/h reached the Stork ‘Zozu’ in the 4 km distant village of Böhringen. Using powered flapping flight, the stork flew against the wind at the time the leaf volatiles were expected to reach it, travelling at very low altitude towards the freshly farmed field. (**B**) Altitude profile and GPS track of the stork flight. Photo by Martin Wikelski, MPIAB.

**Figure 3:**
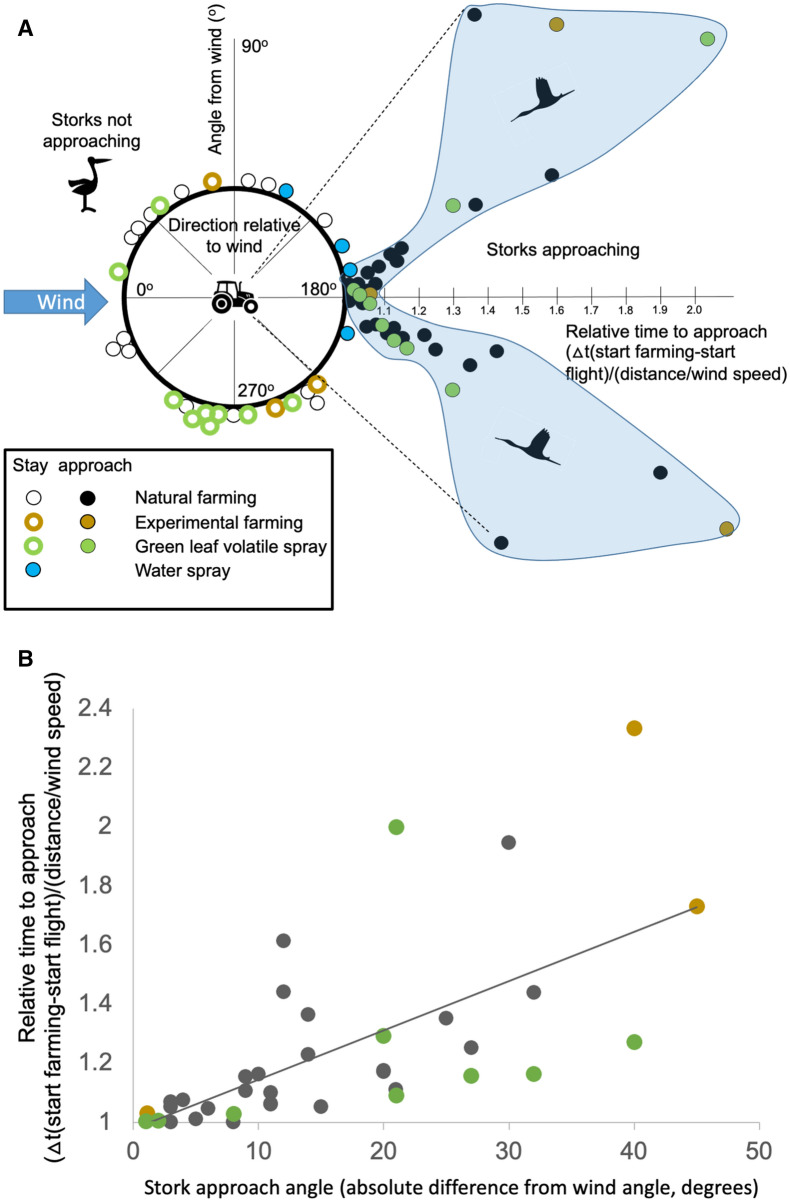
Summary of the reactions of storks towards natural or simulated farming activities in all observations and experiments. For all observations, the prevailing wind direction was standardized towards 180°. (**A**) All dots indicate independent observations of single storks or groups of storks who had visual contact with each other, i.e., stood together in a field or flew together within a flock. The large circle indicates the compass directions towards storks from each respective field. Colored dots indicate different natural or experimental situations. Open circles show storks that stayed at their locations independent of fresh farming activity, closed circles highlight storks (individuals or groups shown as one dot) that approached the farmers. The response time to approach a freshly mown field increases towards the right on the x-axis, which is standardized by wind speed and distance and thus dimensionless for each observation. The blue shaded area encompasses all storks that approached the farming activity. No stork approached freshly farmed fields from an upwind location. The two dotted lines prescribe the angle (ca. ± 45° from downwind) below which all storks except one (open circle) flew towards the odor source. Note that the x-axis is dimensionless ((start farming-start flight)/(distance/wind speed)) and for all filled circles indicates how quickly storks reacted as soon as the wind arrived at their location, presumably carrying the odors. The y-axis indicates the angle where storks were located towards the source of the odor. (**B**) From a downwind cone of ca. ± 45°, storks approached ongoing natural farming activities (black dots), experimental farming (brown dots) or the pure spraying of leaf alcohol (green dots). The line indicates a significant linear regression for all approaches (y = 0.017x + 0.98; R^2^ = 0.41; p < 0.0001).

**Figure 4:**
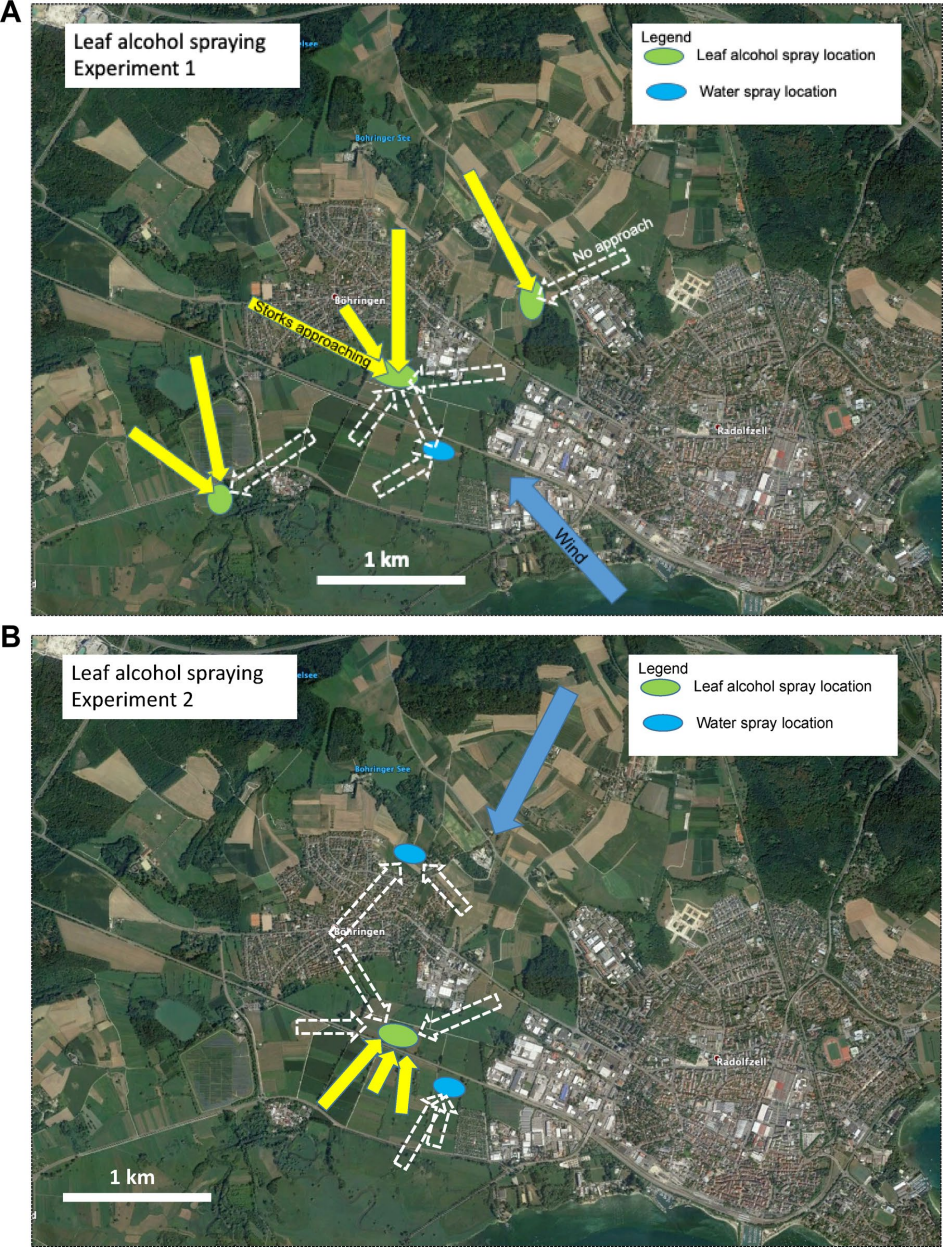

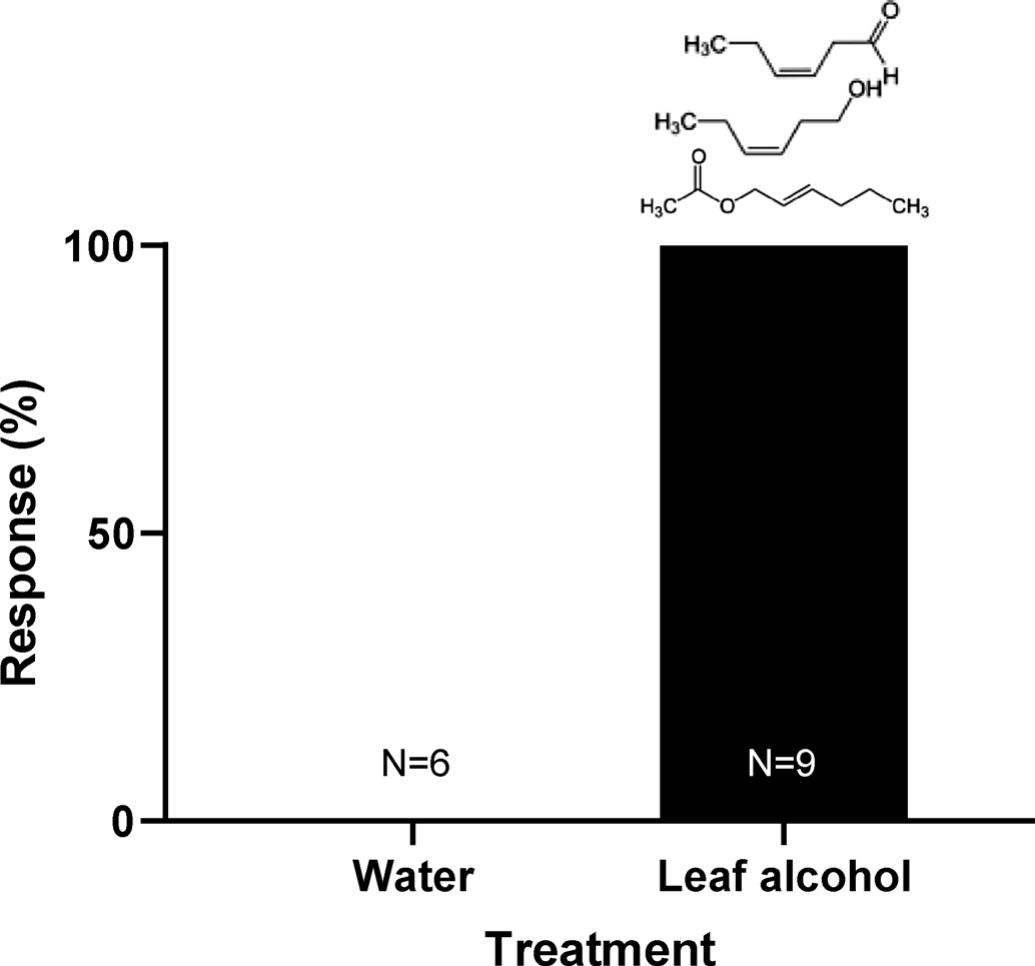
Storks immediately approached an aerially-sprayed volatile organic compound mix. (**A**) and (**B**) Maps of the area around Böhringen, Germany, showing the locations of water spraying (blue area), leaf alcohol spraying (green areas), the downwind approach locations and distances by independently approaching storks (yellow arrows), and upwind areas where storks resided in fields, but did not approach (white stippled arrows). (**A**) Experiment conducted on 27-04-2018, (**B**) Experiment conducted on 02-07-2018. (**C**) Comparison of approach reactions of storks towards water and leaf alcohol. Chemical structures of the three leaf alcohols are indicated above the black bar. The difference between the treatments is highly significant (X^2^ = 11.7, N = 15, df = 2, p = 0.003). Maps were created from Google Earth.

We hypothesized that storks may use downwind chemical cues from farmed fields and only approach when they get these olfactory cues. We observed one entire local population of white storks from a plane during 11 flights lasting 2–4 h to determine where all ca. 70 individual storks are located and how they potentially interact with each other and their environment^31^. During flight observations we determined where all storks were in relation to farming activity within a radius of ca. 6 km (Figs. 2, 4A,B), i.e., knew where storks were residing up- und downwind. To determine whether the storks solely use olfactory cues we had to exclude all other known food-sensing possibilities such as visual, auditory or social cues. Visual cues could be excluded whenever storks had no visual access to farming sites, primarily because trees or the local topography prevented visual information gathering. Auditory cues for farming activities were similarly excluded by the distance to storks on the ground. A more complex task was to make sure storks could not use flying conspecifics or other soaring birds such as kites or buzzards of social indicators for farming activities. Only those instances when aerial observations confirmed that no other bird was indicating the presence of the farming activities were included (black and white dots in Fig. 3).

Aerial observations were conducted from a Cessna 172 plane stationed at Konstanz airport (EDTZ), in approximately 5 min flight distance from the study area. We observed the entire stork population of the village of Böhringen, roughly 70 individuals, as they foraged in the farm fields around this village. From an altitude of 800–1200 m above ground, we could safely observe an area of about 6 km radius and after some initial search, locate all white storks on the ground or in the air. We could also see all farming activity around the village (Fig. S2).

We conducted our observations only in the spring and early summer and on days with clear sky and sunny weather, i.e., when farming/mowing activities were potentially present. We furthermore made sure that no storks were circling higher than about 50 m above the vegetation when we conducted our observation trials. This fact ensured that storks could not rely upon social information from other storks to be alerted to, and thereby socially find farm activities.

Whenever a farmer started to mow a pasture, we first made sure that no kites, buzzards or storks were potentially indicating this field through their aerial flight activities to storks. Such situations sometimes occurred particularly around noon and in the afternoons as thermals allowed the birds to stay high above the fields. We did not include any such observations in our data set. We also ensured that no storks were in the visible range of the farming activity, which was ensured by the fact that either trees or topography prevented any visual information on this farming activity for any stork. Auditory information was also excluded as information system for storks because farming equipment was used in many circumstances (n = 57 aerial observations) when no field was mowed and no storks arrived accordingly (n = 0 arrival of storks during the 57 observations; x^2^-test, p < 0.001). We thus concluded that storks did not use auditory information to approach mowing areas. Nevertheless, we also discounted any observations where storks were closer than 600 m from the farming activity, as we wanted to be certain that no auditory information from this activity could reach the storks. This distance is a conservative estimate for any auditory information from farm vehicles to travel and be received by a regular bird auditory system such as in storks. Our assessment is based upon the conservative assumption that storks have their best hearing within 0.5–15 kHz at a threshold of 4 dB^32^. We then determined whether farming activity could hit this threshold at a distance of 600 m. However, we could not record any auditory information from farming activity against background noise beyond 300 m.

We then circled above a mowed field and observed if any stork would approach from any direction towards the farming activity. As soon as any stork started to fly, we noted down the time and direction, as well as the number of storks in the flock, in case more than one stork started to fly. Subsequently we denoted whether the stork or group of storks approached the farmer, and at what time they settled to search for food in this freshly mowed field. We also noted the location of storks that did not show any reaction towards the farming activity.

In addition to the aerial observations, we also conducted concurrent ground truthing observations to ensure that we did not miss or misinterpret any information from the air. We found no inconsistencies between ground observations and aerial observations. Several of the observed storks were carrying high-definition GPS recording devices and thus the aerial observations could be confirmed and calibrated with onboard recording of the animals. We measured wind speed and direction both in the air through the Garmin G 1000 instrument of the Cessna and on the ground using standard anemometers. Whenever possible, we confirmed the precise wind directions by using smoke from small local fires or dust emerging from faming activities.

### Experimental farming activity and green leaf volatile spraying

To experimentally determine whether storks only use olfactory information to approach potential foraging sites we furthermore performed two experiments: First, we took freshly cut grass from a field some 15 km distant from the experiment site and placed this grass approximately 2.5 km upwind from several spatially separated groups of storks (orange dots in Fig. 3). The second more chemically specific test was to spray a three-component mixture simulating the scent of freshly cut grass over a previously untreated grass covered field from an ultralight plane (green dots in Fig. 3). The chemical mixture designed to simulate the scent of fresh cut grass comprised of three green leaf volatiles (Z)-3-hexenal, (Z)-3-hexenol and hexenyl acetate. These have been shown to be the main emitted volatile organic compounds when leaves are mechanically wounded in laboratory and in field measurements over fresh cut pasture^33,34^.

On September 9, 2017, we conducted an experiment to test whether it would be sufficient for attracting storks to fields is only freshly cut grass were provided. In this case, there would be no ultimate reason for the storks to approach the area because no food items are available for storks if tall grass is not physically cut on site to allow for access to insects or rodents. We arranged for the farmer to cut one entire truckload of fresh grass ca. 15 km away and transport it right away, within 45 min (travel time), to an area roughly 2.5 km distant from areas where storks were standing on the ground. The permission to cut this grass was obtained from the local authorities. The farmer then distributed the freshly cut grass on a field that was cut roughly 2 weeks ago, i.e., where the grass was still very short and no storks would approach to forage under such conditions. As usual, we were conducting aerial and ground observations on this field and the surrounding stork population to determine if any storks would approach from any direction.

To ultimately test whether olfactory information could be the sole cue for storks to approach freshly farmed fields, we used a homogenous aqueous mix of the three main green leaf volatiles ((Z)-3-hexenal, (Z)-3-hexenol, hexenyl acetate; W256102, W256323, W317100, respectively, from Sigma-Aldrich, Germany), sprayed into the air from a low-flying ultralight plane (Video S1). We prepared an electronic garden spray pump (10 L content) and attached this safely to the passenger seat of the ultralight. We then flew the ultralight to an area with no farming activity and in very low, i.e., ca. 20 m altitude overflights of a field, sprayed these green leaf alcohol-in-water suspensions into the air. Again, we conducted additional aerial observations from the Cessna plane to determine whether any storks would approach or if storks were not reacting to the chemicals in the air.

In our calculations of spray concentrations, we tried to approximate the natural concentrations of leaf volatile compounds in the air above a mowed field. However, we did not quantify these concentrations as it is currently ultimately impossible to determine quantitatively what concentrations of leaf volatiles are inhaled and perceived by individual birds in the field. We used the following concentrations of green leaf volatiles: We determined that we needed to spray an area roughly the size of 50 × 100 m to realistically represent a minimum farming, i.e., field mowing activity. If we assume we spray 2 mL/m^2^, then the overall amount of solution to be sprayed would be 10 L, one tank of aqueous solution to be carried in the ultralight plane. We determined that a mixture of 1 ppm would be generously realistic, such that 1 mL (of each of the three pure leaf volatile compounds) should suffice. The resulting smell in the air and in an underlying field qualitatively represented the natural mowing activity of farmers as perceived by human observers during test trials. Therefore, we expected that our experimental leaf volatile spraying adequately represented near-natural levels of mowing smell.

### Statistics

We calculated statistical tests using IBM SPSS Statistics for Mac, release 27.0.1.0.

## Results

To reach the foraging area, individuals flew by powered flight only a few meters above the terrain as soon as the air from the farmed fields reached them (Fig. 3B). An example is shown for stork ‘Zozu’ (Fig. 2) which was tagged with a high-definition GPS tag. The on-animal data confirm the aerial observations and show that no visual or auditory information was available over the ca. 4 km approach distance. As Zozu was the first bird to arrive at the farming site, no other birds could have indicated this potential foraging location.

In general, when examining natural farming events, we observed that storks who resided upwind from a freshly farmed field did not approach the test area (Fig. 3). In contrast, only storks located in a downwind cone of ca. 75° approached the farming activity (black dots in Fig. 3A). The further off-centerline of the wind direction the storks were, the slower they approached the test area (Fig. 3B). This relationship held for natural observations, as well as for the experiments employing displaced grass and for the experimental spraying of the green leaf volatiles.

Storks not only approached fields from downwind during natural farming events, but also when a complete trailer of freshly cut grass, obtained from a 15 km far-away location, was transported and unloaded within 45 min after mowing on a non-cut field. The wind speed during this experiment was 7 km/h. The 3 independently approaching individuals or groups of storks landed and started to search for food, but after ca. 10 min realized that no food was available within the cut grass. The storks then left again (Fig. S1). In a final step, to unambiguously test whether the chemical signals alone, emanating from the cut grass, attracted the storks, in 4 independent trials we sprayed a leaf alcohol mix simulating naturally freshly cut grass from an ultralight plane flying very low (20 m) above a non-farmed field (Video S1). We monitored an area of 5 × 6 km for all storks (49 on 27-04-2018, 37 on 02-07-2018).The cut grass scent cocktail attracted 9 independently approaching individuals or groups of storks from locations that were 0.5 to 1.2 km away (Fig. 4A,B), whereas the ultralight plane alone, or water sprayed from the ultralight, did not elicit any reactions in storks residing within the same or shorter distances (Fig. 4C; see X^2^-test, p = 0.003). Storks that were not within a downwind cone of ± 45° of the leaf alcohol spraying did not approach the area (n = 7). This is consistent with their natural approach angles to farmed areas (Fig. 3A).

## Discussion

We show that storks only approach cut pastures upwind when they are able to perceive atmospheric information from a freshly farmed field, but do not rely upon auditory or visual, including social, cues. We experimentally demonstrate that cut grass alone, and even the three main olfactory components of cut grass (green leaf volatiles), are sufficient to attract storks to approach supposed foraging sites. This unambiguous demonstration of the use of atmospheric odors to learn about foraging opportunities breaks with the notion that storks, like most birds, primarily use vision to find food.

While for most animals it is demonstrated and accepted that odors guide much of their foraging behavior^35^, diurnally active birds, i.e., the majority of the ca. 10K species avian clade, were perceived to be a notable exception (except for New-world vultures and some seabirds)^36^. The advanced use of atmospheric circulation models^37^, introduced some two decades ago, has provided a new insight into the potential for large-scale olfactory connectivities across local regions. Since then, it is now generally accepted that homing pigeons^18^ and perhaps other long-distance migratory animals^17,30^) use environmental odor information for navigation—a finding that was heavily debated for the previous four decade^38,39^. Similarly, research in the ecological aspects of environmental odors in vertebrates and birds in particular has increased strongly^9,14,20,26,30,40,41^. The decade-long debate about odor-use in birds shows how strongly behavioral researchers have been biased by their own perceptions of the environment as well as earlier dogmatic notions that olfaction plays no major role as sensory input for most birds^36^. Instead, we suggest that the use of olfaction in avian food search may be more prevalent than previously expected.

## Supplementary Information

Supplementary Information 1.

Supplementary Video S1.

## Data Availability

Data are available from Movebank data repository, http://www.datarepository.movebank.org, under DOI (available upon publication).
