# Supplementary material for: Smell of green leaf volatiles attracts white storks to freshly cut meadows
Source: Sci Rep. 2021 Jun 18;11:12912. doi: 10.1038/s41598-021-92073-7 (PMC8213700; doi:10.1038/s41598-021-92073-7)
Supplement: Supplementary file 1 — Supplementary Information 1. [file 41598_2021_92073_MOESM1_ESM.docx]

Supplementary Materials for

Smell of green leaf volatiles attracts White storks to freshly cut meadows

Martin Wikelski, Michael Quetting, Yachang Cheng, Wolfgang Fiedler, Andrea Flack, Anna Gagliardo, Reyes Salas, Nora Zannoni, Jonathan Williams.

Correspondence to: wikelski@ab.mpg.de

**This PDF file includes:**

Figs. S1 to S2

Captions for Movie S1

**Other Supplementary Materials for this manuscript include the following:**

Movie S1


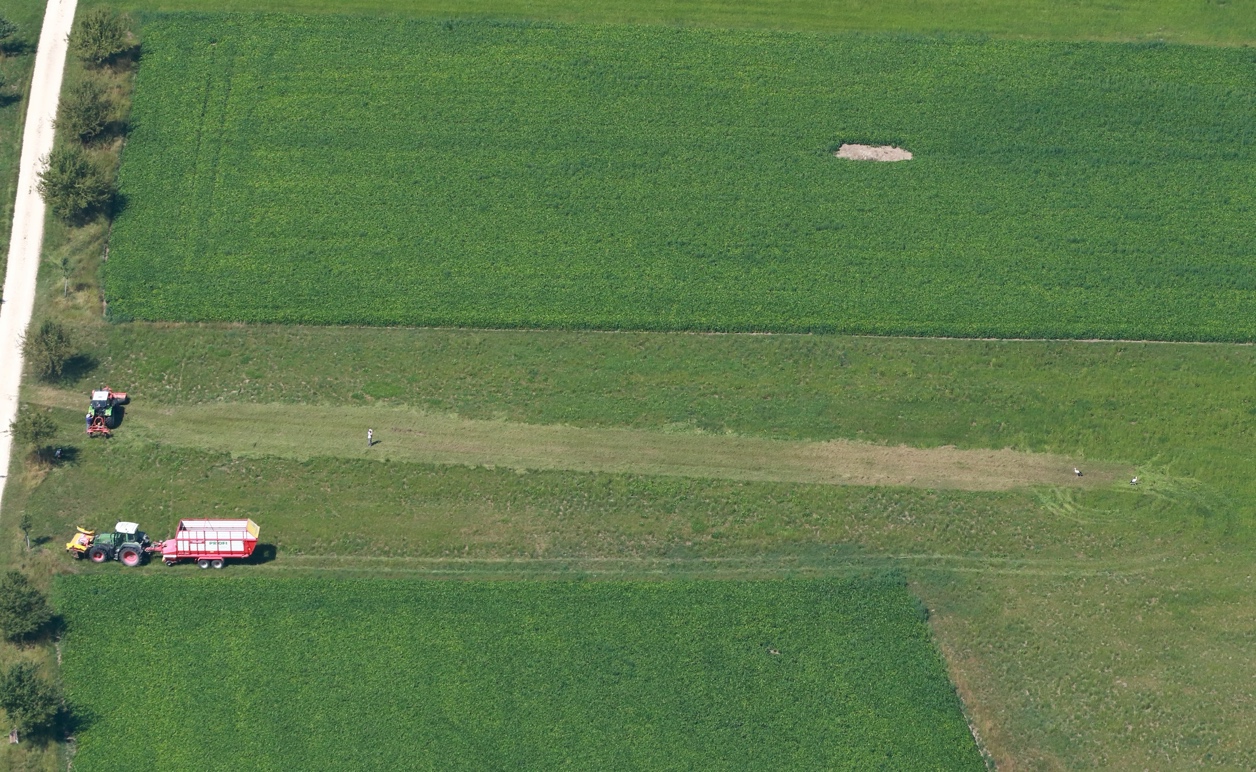


Fig. S1.

Aerial picture of the experimental farming activity. The tractor below was used to cut the grass in ca. 15km distance and transport it to the current field. The other tractor was used to swirl the cut grass into the air and free the chemical compounds (green leaf alcohols primarily) that might attract storks. Please note the ground observer standing on the experimentally distributed cut grass, as well as two White storks who already approached and landed on the field. These storks were walking up and down the area with the cut grass, but did not feed. The storks left again after ca. 10 minutes, i.e., when not finding food items in this field. Other storks from other locations arrived subsequently and also behaved in the same way. Photo by Christian Zieger, MPIAB.


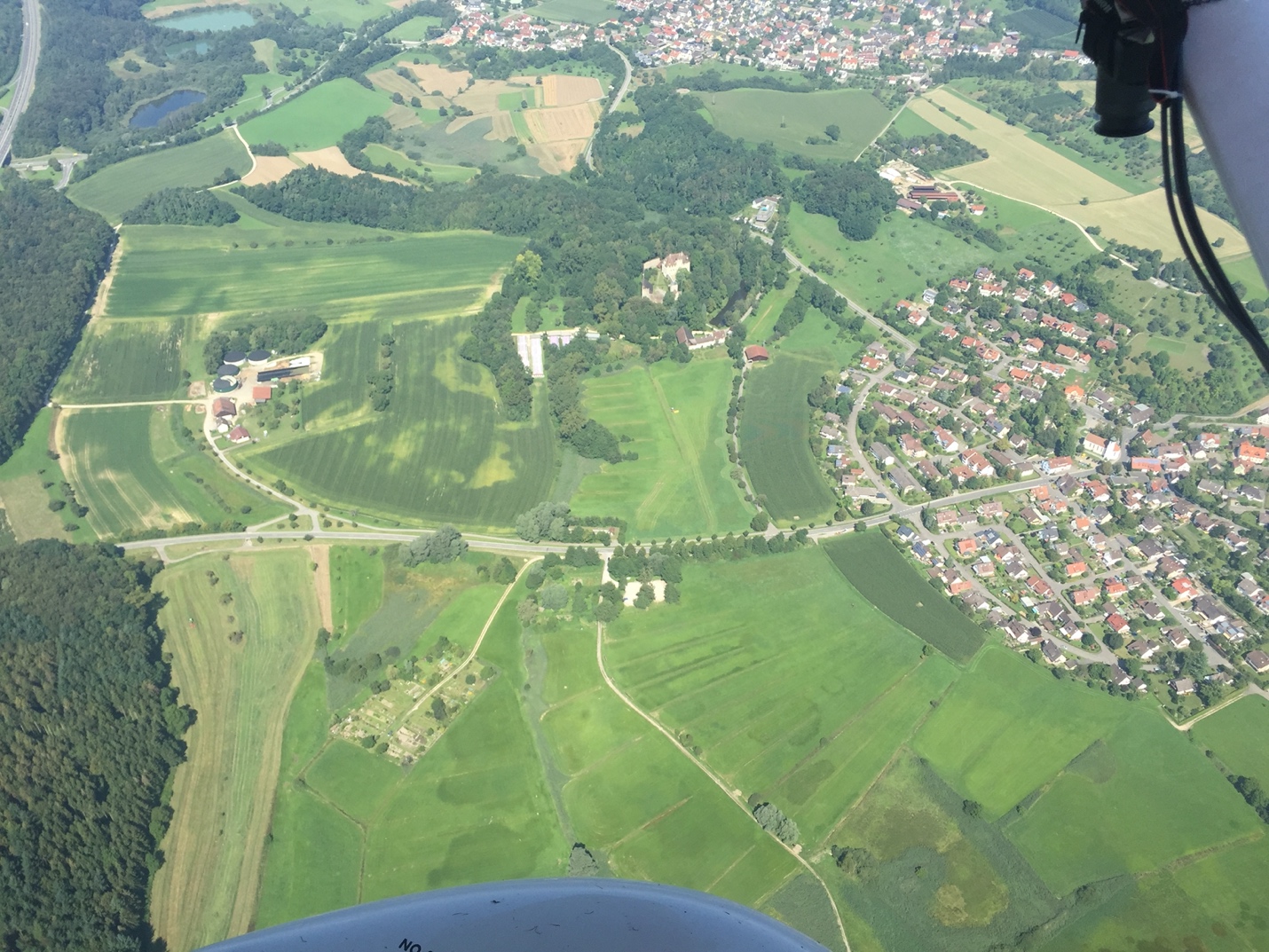


Fig. S2.

A typical view from the Cessna plane during the observations. Please note the farming activity in the lower left part of the picture as well as the White storks on the ground in this field. Photo by Martin Wikelski, MPIAB.

Movie S1.

Movie showing the ultralight plane in the air during the green leaf volatile spraying campaign, as well as storks approaching this area.
